# Supplementary material for: Metabolic and proteomic signatures of type 2 diabetes subtypes in an Arab population
Source: Nat Commun. 2022 Nov 19;13:7121. doi: 10.1038/s41467-022-34754-z (PMC9675829; doi:10.1038/s41467-022-34754-z)
Supplement: Supplementary file 7 — Reporting Summary [file 41467_2022_34754_MOESM7_ESM.pdf]

nature portfolio

Corresponding author(s): Karsten Suhre

Last updated by author(s): Oct 11, 2022

Reporting Summary

Nature Portfolio wishes to improve the reproducibility of the work that we publish. This form provides structure for consistency and transparency in reporting. For further information on Nature Portfolio policies, see our Editorial Policies and the Editorial Policy Checklist.

Statistics

For all statistical analyses, confirm that the following items are present in the figure legend, table legend, main text, or Methods section.

n/a	Confirmed	
☐	☒	The exact sample size (*n*) for each experimental group/condition, given as a discrete number and unit of measurement
☐	☒	A statement on whether measurements were taken from distinct samples or whether the same sample was measured repeatedly
☐	☒	The statistical test(s) used AND whether they are one- or two-sided<br>*Only common tests should be described solely by name; describe more complex techniques in the Methods section.*
☐	☒	A description of all covariates tested
☐	☒	A description of any assumptions or corrections, such as tests of normality and adjustment for multiple comparisons
☐	☒	A full description of the statistical parameters including central tendency (e.g. means) or other basic estimates (e.g. regression coefficient) AND variation (e.g. standard deviation) or associated estimates of uncertainty (e.g. confidence intervals)
☐	☒	For null hypothesis testing, the test statistic (e.g. *F*, *t*, *r*) with confidence intervals, effect sizes, degrees of freedom and *P* value noted<br>*Give P values as exact values whenever suitable.*
☒	☐	For Bayesian analysis, information on the choice of priors and Markov chain Monte Carlo settings
☒	☐	For hierarchical and complex designs, identification of the appropriate level for tests and full reporting of outcomes
☐	☒	Estimates of effect sizes (e.g. Cohen's *d*, Pearson's *r*), indicating how they were calculated

Our web collection on statistics for biologists contains articles on many of the points above.

Software and code

Policy information about availability of computer code

Data collection	Standard database software used by the Qatar Biobank (MS SQL Server 2008 R2).
Data analysis	Standard statistical analysis was carried out using functions implemented in R Studio version 4.0.5 and version 1.4.1106. Documentation on how to use the standard built-in R functions can be found at https://www.rstudio.com/products/rpackages/.

For manuscripts utilizing custom algorithms or software that are central to the research but not yet described in published literature, software must be made available to editors and reviewers. We strongly encourage code deposition in a community repository (e.g. GitHub). See the Nature Portfolio guidelines for submitting code & software for further information.

Data

Policy information about availability of data

All manuscripts must include a data availability statement. This statement should provide the following information, where applicable:

- Accession codes, unique identifiers, or web links for publicly available datasets
- A description of any restrictions on data availability
- For clinical datasets or third party data, please ensure that the statement adheres to our policy

The informed consent given by the study participants does not cover posting of participant level phenotype data in public databases. However, data are available in an MS SQL Server 2008 R2 database, upon request from Qatar Biobank (QBB) (https://www.qatarbiobank.org.qa/research/how-to-apply). Requests are submitted online and are subject to approval by the QBB board.
The custom-design Novartis SOMAscan is available through a collaboration agreement with the Novartis Institutes for BioMedical Research (lori.jennings@novartis.com). Data from the AGES Reykjavik study are available through collaboration (AGES_data_request@hjarta.is) under a data usage agreement with the Icelandic Heart Association.
The QMDiab data are available under restricted access for the informed consent given by the study participants does not cover posting of participant level phenotype data in public databases. Access can be obtained in the form of an R data file, upon request from the corresponding author.
Data from the ANDIS study are available upon request from the ANDIS steering committee (emma.ahlqvist@med.lu.se).

Field-specific reporting

Please select the one below that is the best fit for your research. If you are not sure, read the appropriate sections before making your selection.

- [x] Life sciences
- [ ] Behavioural & social sciences
- [ ] Ecological, evolutionary & environmental sciences

For a reference copy of the document with all sections, see nature.com/documents/nr-reporting-summary-flat.pdf

Life sciences study design

All studies must disclose on these points even when the disclosure is negative.

Sample size	Qatar Biobank (QBB) includes a population of Qatar nationals or long-term residents (≥15 years living in Qatar), aged 18 years and older in the State of Qatar. At the time of analysis, QBB data was available for 6,218 participants.
Data exclusions	429 participants with incomplete records and 894 individuals with HbA1c ranging between 5.7 and 6.4 who did not match our diabetes definition were excluded, leaving 4,895 samples for analysis.
Replication	All of the following replications were performed once and independently: 1- Replication of association of T2D with proteomics. Association data for the replication of the T2D protein association was obtained from the published AGES study.<br>2- Replication of association of T2D with metabolomics. Metabolomics associations with T2D have been previously reported for the QMDiab study using the Metabolon HD2 platform.<br>3- Replication of associations of T2D subtypes with proteomics. Replication of the subgroup specific proteins was attempted in the ANDIS study.<br>Details for each replication experiment (number of successful replications) are mentioned in the manuscript.
Randomization	N/A. This is a population study, so participants were collected at random.
Blinding	N/A. This is a population study, so blinding of samples is not applicable. Blinding is applicable when there is group allocation from one or more individuals involved in a clinical research study, such as a randomized controlled trial. Here, we do not allocate any individuals to any particular groups because it is a population study where we study both common and subject-specific characteristics.

Reporting for specific materials, systems and methods

We require information from authors about some types of materials, experimental systems and methods used in many studies. Here, indicate whether each material, system or method listed is relevant to your study. If you are not sure if a list item applies to your research, read the appropriate section before selecting a response.

Materials & experimental systems

n/a	Involved in the study	
☒	☐	Antibodies
☒	☐	Eukaryotic cell lines
☒	☐	Palaeontology and archaeology
☒	☐	Animals and other organisms
☐	☒	Human research participants
☐	☒	Clinical data
☒	☐	Dual use research of concern

Methods

n/a	Involved in the study	
☒	☐	ChIP-seq
☒	☐	Flow cytometry
☒	☐	MRI-based neuroimaging

Human research participants

Policy information about studies involving human research participants

Population characteristics	Qatar Biobank (QBB) includes a population of Qatar nationals or long-term residents (≥15 years living in Qatar), aged 18 years and older in the State of Qatar. Extensive baseline socio-demographic data, clinical and behavioral phenotypic data and serum concentrations of HbA1c, triglycerides, glucose, C-peptide, creatinine, total cholesterol, LDL-C and HDL-C, and multiple other clinical biochemistry parameters 29 have been measured at the central laboratory of Hamad Medical Corporation<br>(HMC), accredited by the College of American Pathologists. At the time of analysis, QBB data was available for 6,218 participants. Over 96% of the participants reported having grandparents that were Qatari nationals. For 2,155 individuals, metabolomics and proteomics data had been collected in parallel. 429 participants with incomplete records and 894 individuals with HbA1c ranging between 5.7 and 6.4 who did not match our diabetes definition (see below) were excluded, leaving 4,895 samples for analysis. Blood samples were collected more than 2 hours after their last meal or calorie-containing drink in 77% of participants. 50.7% of the participants (52.8% of the T2D cases and 40.6% of the controls) had been fasting for over 8 hours. This dataset was split into two groups, using the samples without omics data as a training set for the clustering (N=2,740), and the samples with omics data as a testing set for validation, and to further evaluate the associations of the metabolite and protein levels with T2D in a case-control setting and with T2D subtypes (N=2,155).<br>The group without omics data contained 631 individuals with T2D which were used to define the cluster coordinates. The group with omics data contained 420 individuals with T2D and was used for cluster validation, and then further for metabolomics and proteomics associations analyses. Demographics of the QBB diabetes and control groups are listed in Table 1 in the manuscript. the mean age for the diabetes group is 51.2 (sd=11.3) and the controls is 34.8 (sd=10.4). The percentage of males is 42.2% in the diabetes group and 34.5% in the controls group.
Recruitment	Recruitment was based on meeting the following criteria: being Qatar nationals or long-term residents (≥15 years living in Qatar), aged 18 years and older in the State of Qatar. Recruitment in Qatar Biobank was completely voluntary.
Ethics oversight	The study was approved by HMC ethics committee and the QBB institutional review board.

Note that full information on the approval of the study protocol must also be provided in the manuscript.

Clinical data

Policy information about clinical studies

All manuscripts should comply with the ICMJEguidelines for publication of clinical research and a completedCONSORT checklist must be included with all submissions.

Clinical trial registration	*Provide the trial registration number from ClinicalTrials.gov or an equivalent agency.*
Study protocol	*Note where the full trial protocol can be accessed OR if not available, explain why.*
Data collection	*Describe the settings and locales of data collection, noting the time periods of recruitment and data collection.*
Outcomes	*Describe how you pre-defined primary and secondary outcome measures and how you assessed these measures.*
