# Supplementary material for: Metabolic and proteomic signatures of type 2 diabetes subtypes in an Arab population
Source: Nat Commun. 2022 Nov 19;13:7121. doi: 10.1038/s41467-022-34754-z (PMC9675829; doi:10.1038/s41467-022-34754-z)

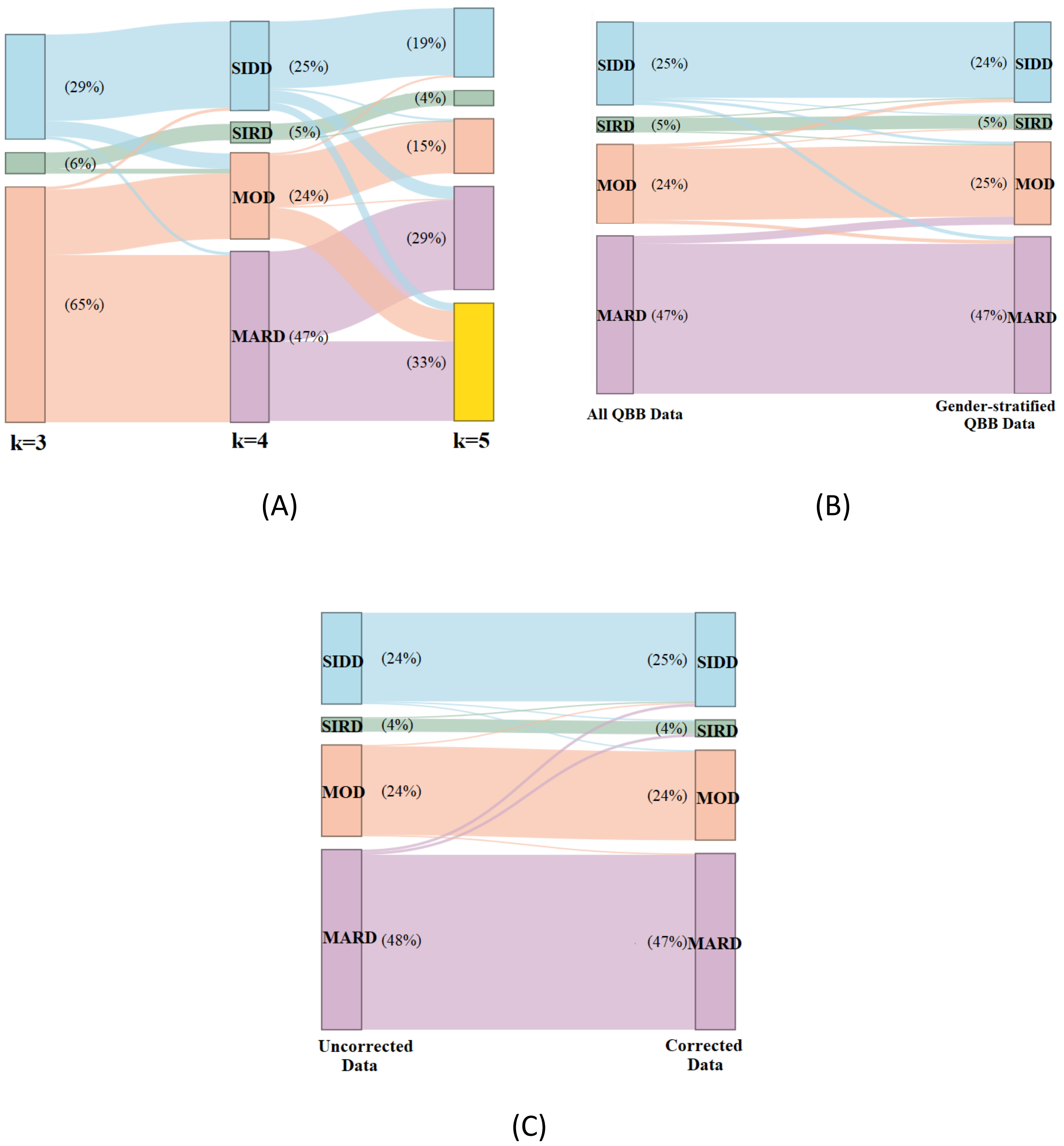

Supplementary Figure 1. Sankey diagrams showing changes in cluster assignment for various scenarios. A) The cluster assignments slightly changed when clustering QBB data using different k (3,4, and 5). To a large extent, clusters 1 and 2 kept the same cluster label using different k, while cluster 3 split into clusters 4 and 5, at k=4 and k=5 respectively. B) Males and females were pooled, then clustered separately. Minor changes in the cluster assignments (7%) were observed when clustering the full data set compared to clustering the gender specific datasets. C) When clustering using uncorrected vs. corrected HOMA variables, most individuals retained the same cluster assignment using the fasting time - corrected data. SIDD: Severe Insulin Deficient Diabetes, SIRD: Severe Insulin Resistant Diabetes, MOD: Mild Obesity-related Diabetes, MARD: Mild Age-related Diabetes

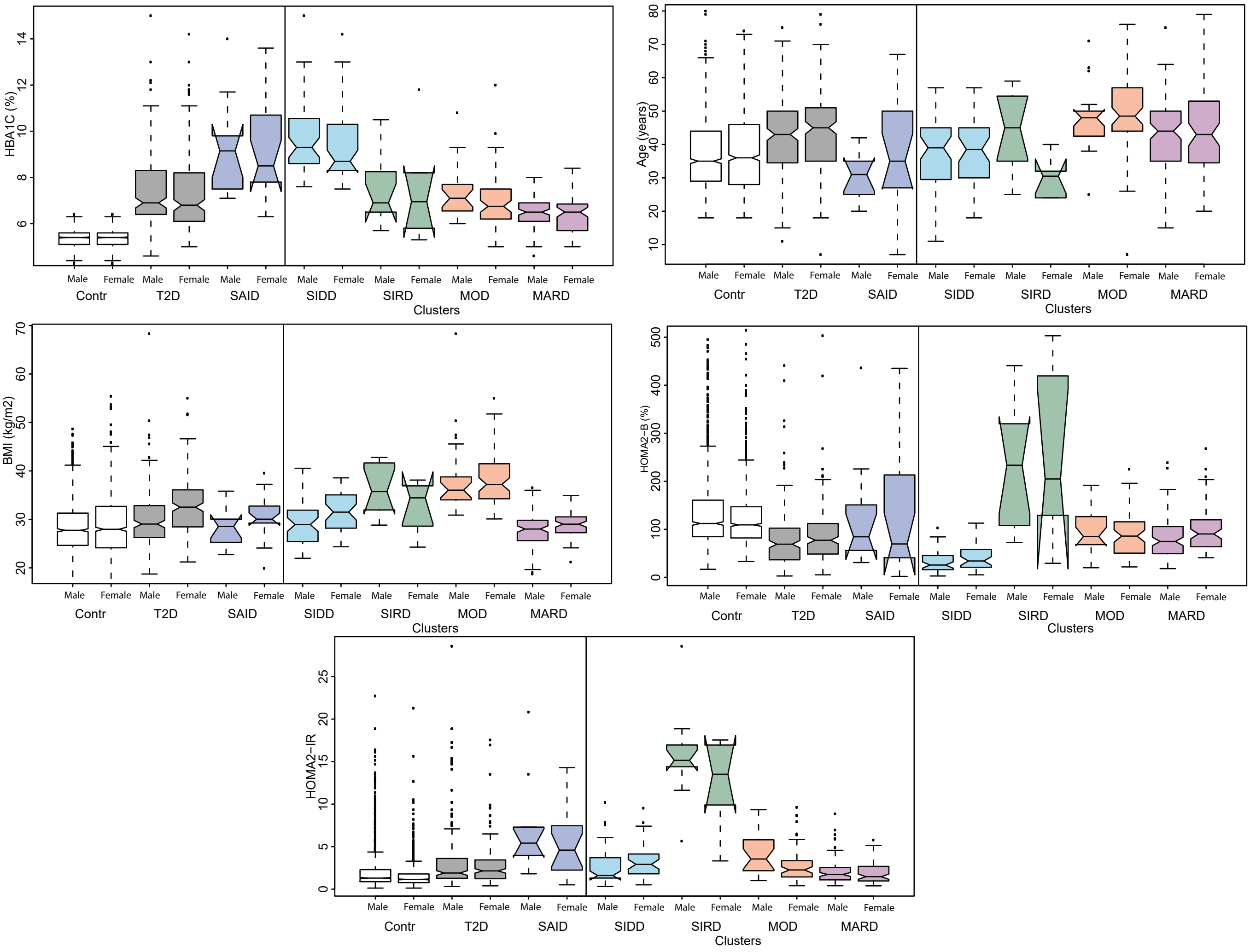

Supplementary Figure 2. Average QBB cluster values stratified by gender. The average cluster values for the five clinical variables (HBA1c, Age, BMI, HOMA2-B, and HOMA2-IR) are comparable between males and females. Contr: Controls (N=1,735 individuals), T2D: Type 2 Diabetes (N=420 individuals), SAID: severe autoimmune diabetes (N=29 individuals), SIDD: Severe Insulin Deficient Diabetes (N=93 individuals), SIRD: Severe Insulin Resistant Diabetes (N= 17 individuals), MOD: Mild Obesity-related Diabetes (N=89 individuals), MARD: Mild Age-related Diabetes (N=177 individuals). Data in boxplots are presented as follows: lower and upper whiskers represent the minima and maxima respectively, box centers represent the median values, bounds of boxes represent the first and third quartiles, notches represent the 95% confidence interval of the median, and dots represent outliers.

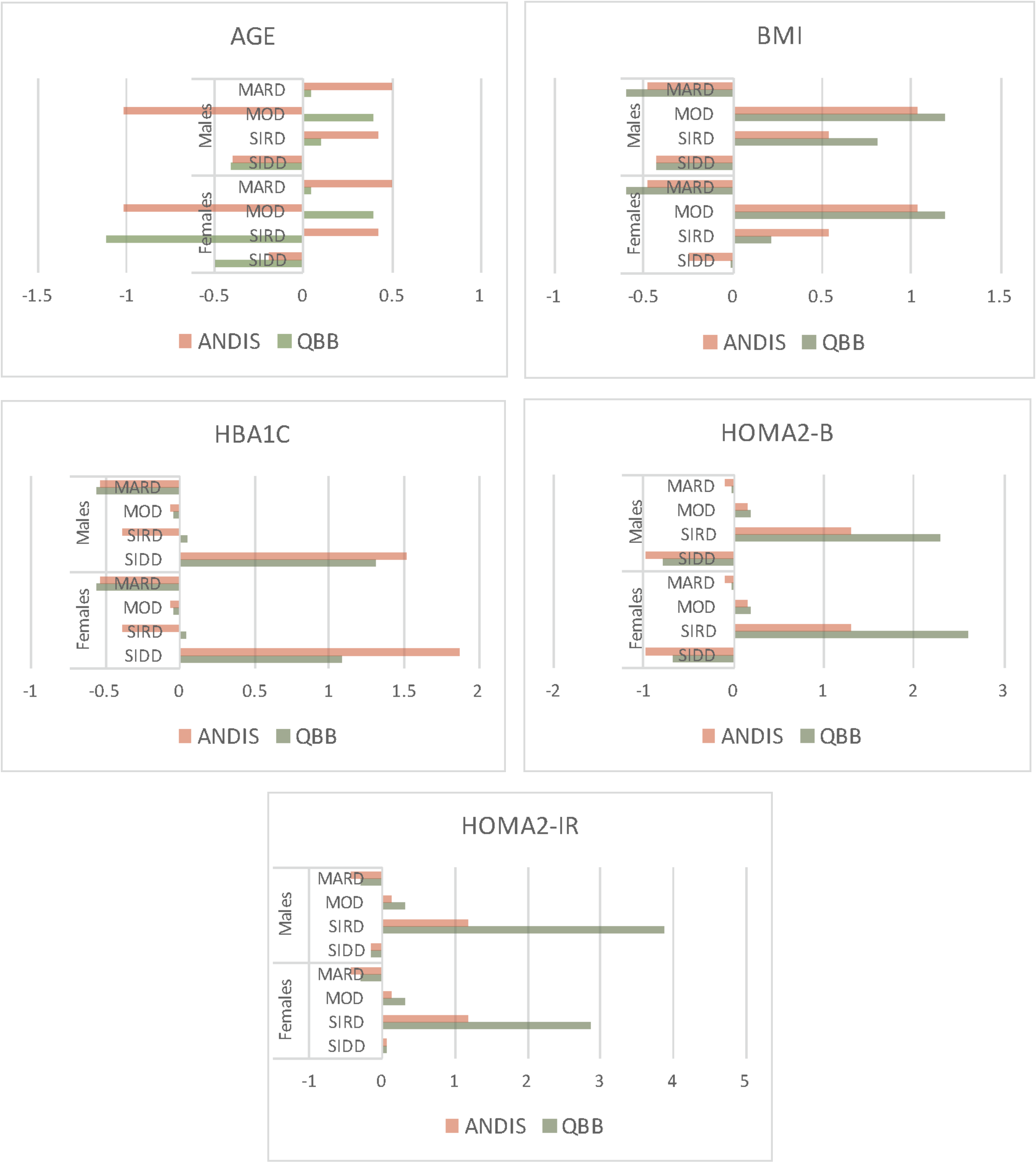

Supplementary Figure 3. Comparison of cluster coordinates for cluster allocations between QBB and ANDIS. Gender-specific variables including age, BMI, HbA1c, HOMA2-B, and HOMA2-IR are shown for the SIDD, SIRD, MOD, and MARD diabetes subtypes. Differences between QBB and ANDIS were mainly observed in the age variable. SIDD: Severe Insulin Deficient Diabetes, SIRD: Severe Insulin Resistant Diabetes, MOD: Mild Obesity-related Diabetes, MARD: Mild Age-related Diabetes. SIDD: Severe Insulin Deficient Diabetes, SIRD: Severe Insulin Resistant Diabetes, MOD: Mild Obesity-related Diabetes, MARD: Mild Age-related Diabetes.

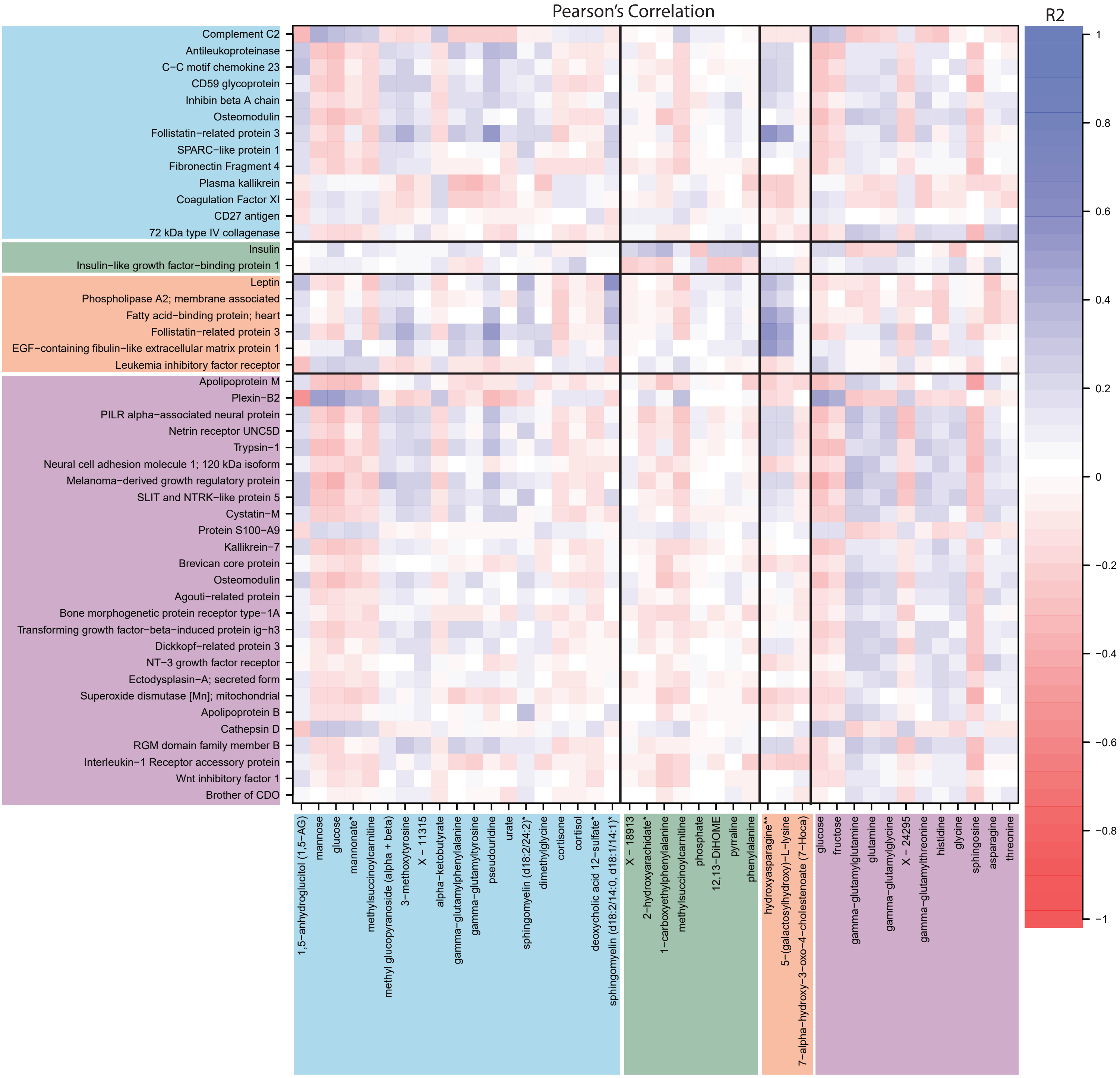

Supplementary Figure 4. Heatmap of the correlations between the identified cluster-specific proteins (y-axis) and the cluster-specific metabolites (x-axis). Correlation coefficients are computed using Pearson's correlation. The proteins and metabolites are color-shaded by the relevant subtype they are associated with (SIDD, SIRD, MOD, and MARD) as used throughout the manuscript. SIDD: Severe Insulin Deficient Diabetes, SIRD: Severe Insulin Resistant Diabetes, MOD: Mild Obesity-related Diabetes, MARD: Mild Age-related Diabetes.

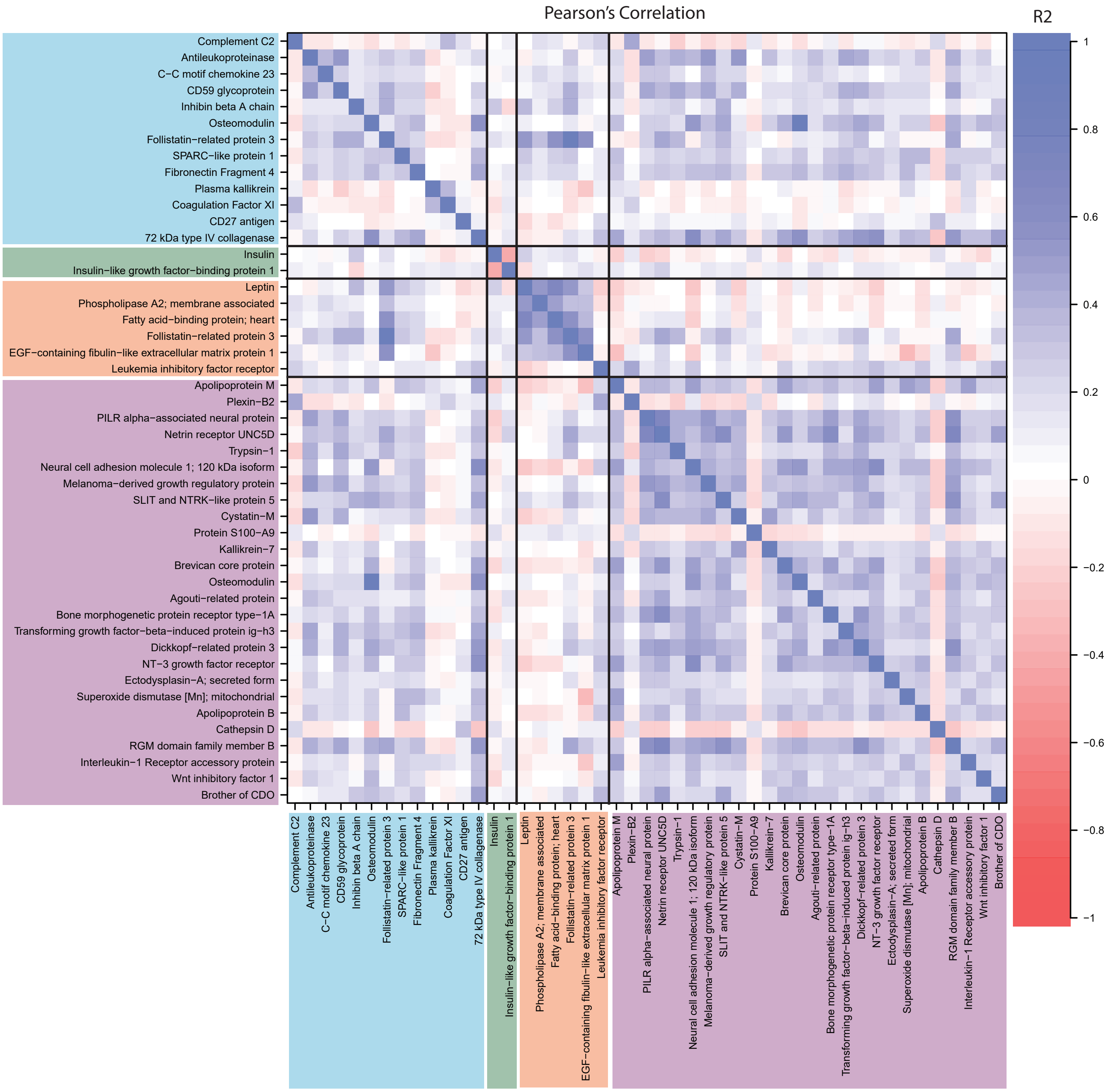

Supplementary Figure 5. Heatmap of the correlations across the identified cluster-specific proteins (both x-axis and y-axis). Correlation coefficients are computed using Pearson's correlation. The proteins are color-shaded by the relevant subtype they are associated with (SIDD, SIRD, MOD, and MARD) as used throughout the manuscript. SIDD: Severe Insulin Deficient Diabetes, SIRD: Severe Insulin Resistant Diabetes, MOD: Mild Obesity-related Diabetes, MARD: Mild Age-related Diabetes.

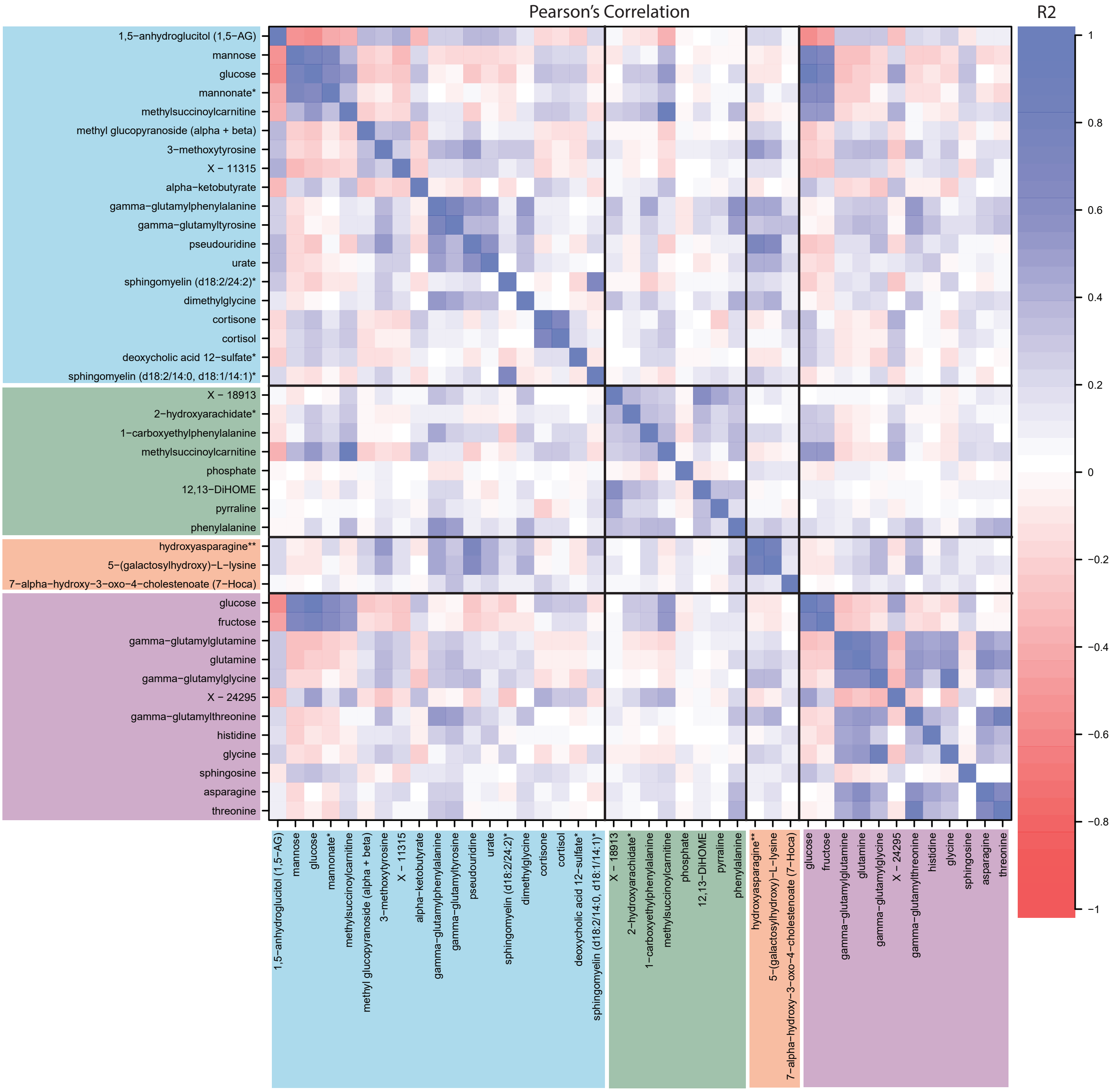

Supplementary Figure 6. Heatmap of the correlations across the identified cluster-specific metabolites (both x-axis and y-axis). Correlation coefficients are computed using Pearson's correlation. The metabolites are color-shaded by the relevant subtype they are associated with (SIDD, SIRD, MOD, and MARD) as used throughout the manuscript. SIDD: Severe Insulin Deficient Diabetes, SIRD: Severe Insulin Resistant Diabetes, MOD: Mild Obesity-related Diabetes, MARD: Mild Age-related Diabetes.

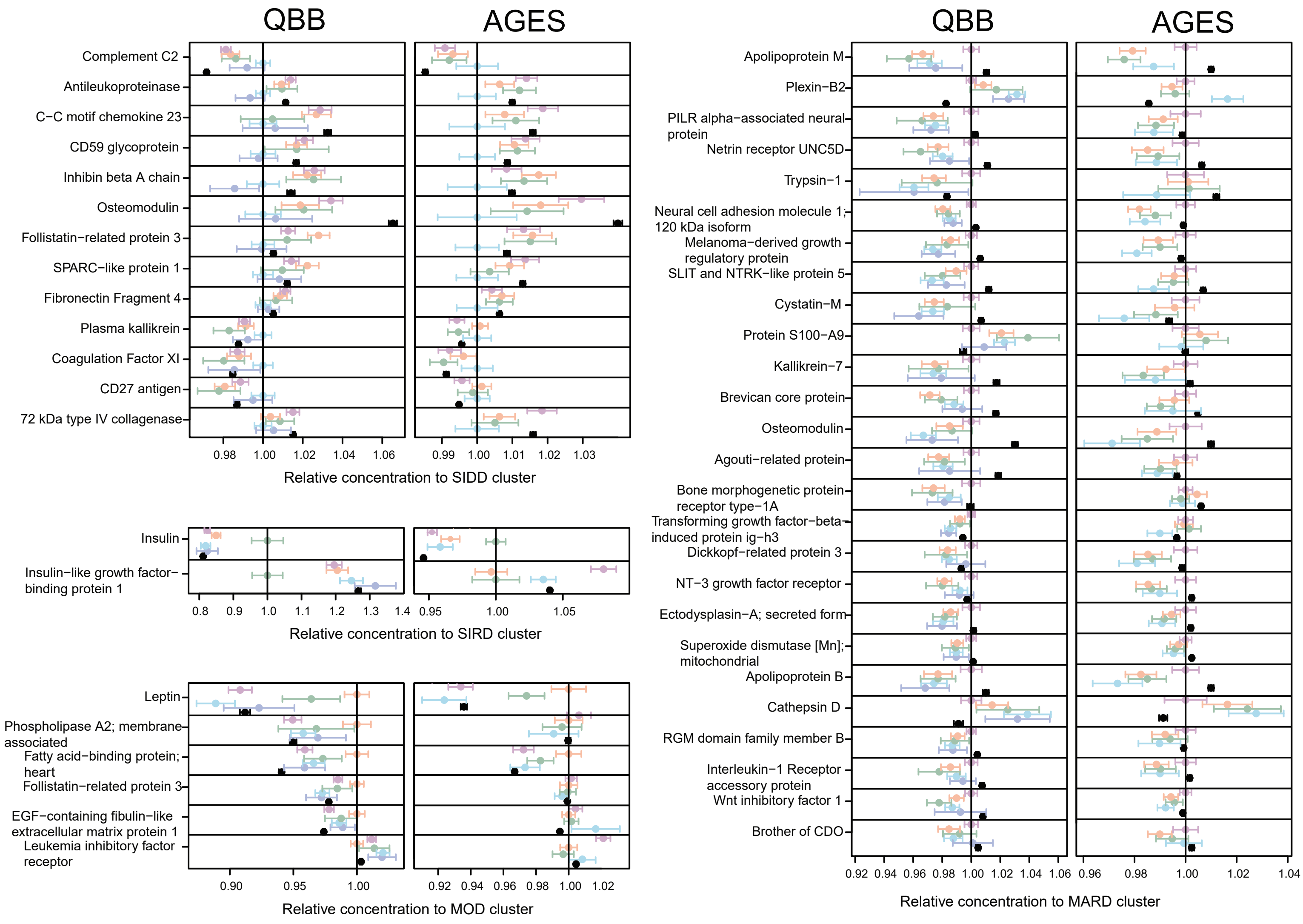

Supplementary Figure 7. AGES study (N=588 individuals) replication of proteins that distinguish individual diabetes subtypes in QBB (N=420). The dots and bars represent the mean protein values and the 95% confidence intervals of the means for proteins that are different in one of the four T2D subtypes compared to all others. Values are normalized by the mean of the respective reference subtype. Data for SAID and the control group are shown for reference. SIDD: Severe Insulin Deficient Diabetes, SIRD: Severe Insulin Resistant Diabetes, MOD: Mild Obesity-related Diabetes, MARD: Mild Age-related Diabetes.

Supplementary Figure 8. Comparison of regression coefficients for subtype-specific proteins between QBB and AGES. Most of the associations (96%) were directionally concordant.

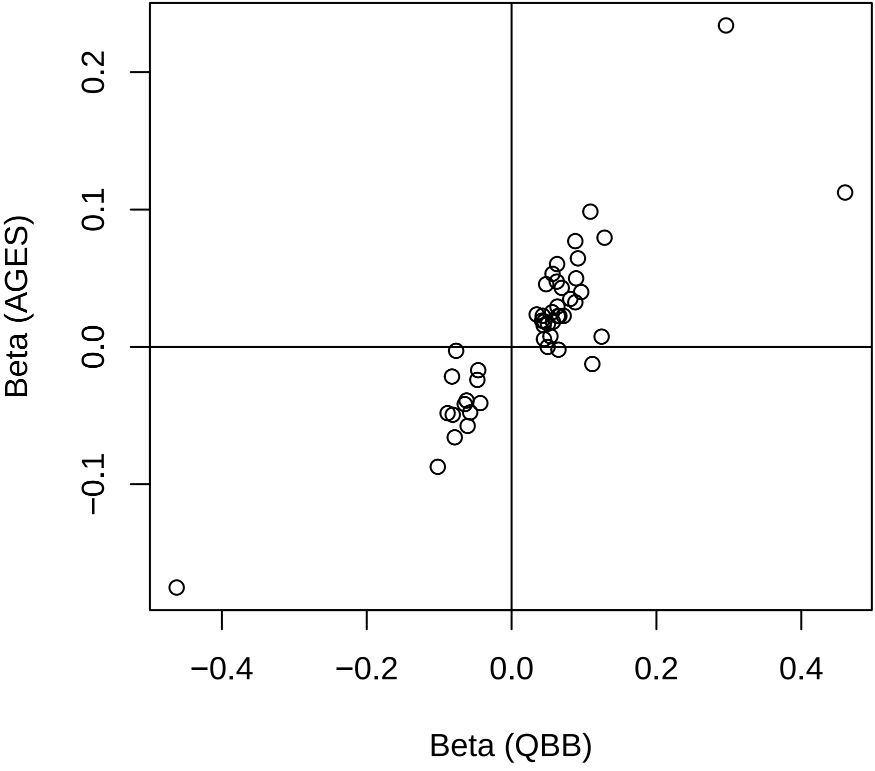

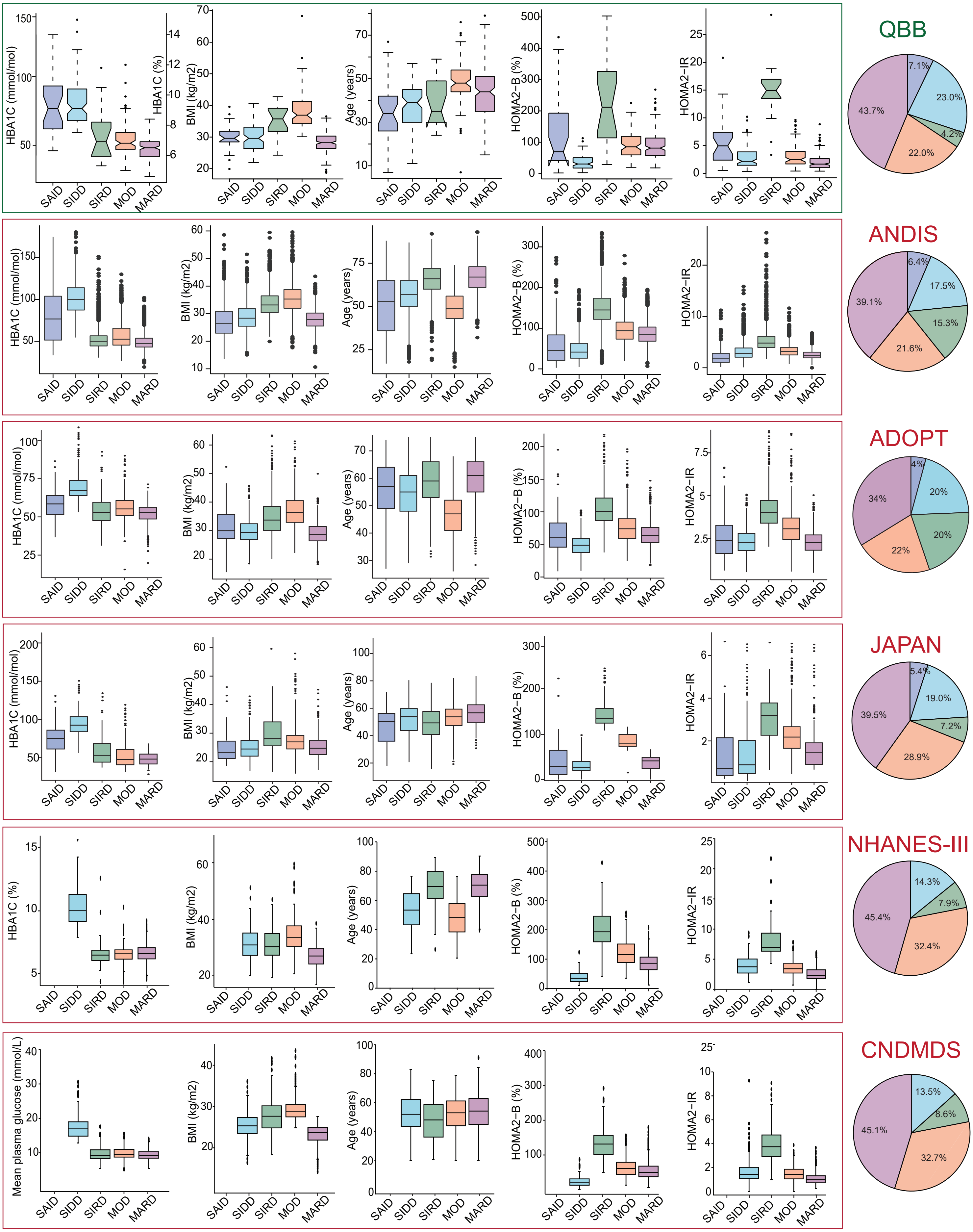

Supplementary Figure 9. Comparison of cluster variables across studies. The cluster variables were similar across many ethnicities (Arab, Scandinavian, British, Japanese, American, and Chinese). SAID: severe autoimmune diabetes, SIDD: Severe Insulin Deficient Diabetes, SIRD: Severe Insulin Resistant Diabetes, MOD: Mild Obesity-related Diabetes, MARD: Mild Age-related Diabetes. Data in boxplots are presented as follows: lower and upper whiskers represent the minima and maxima respectively, box centers represent the median values, bounds of boxes represent the first and third quartiles, and circles represent outliers.

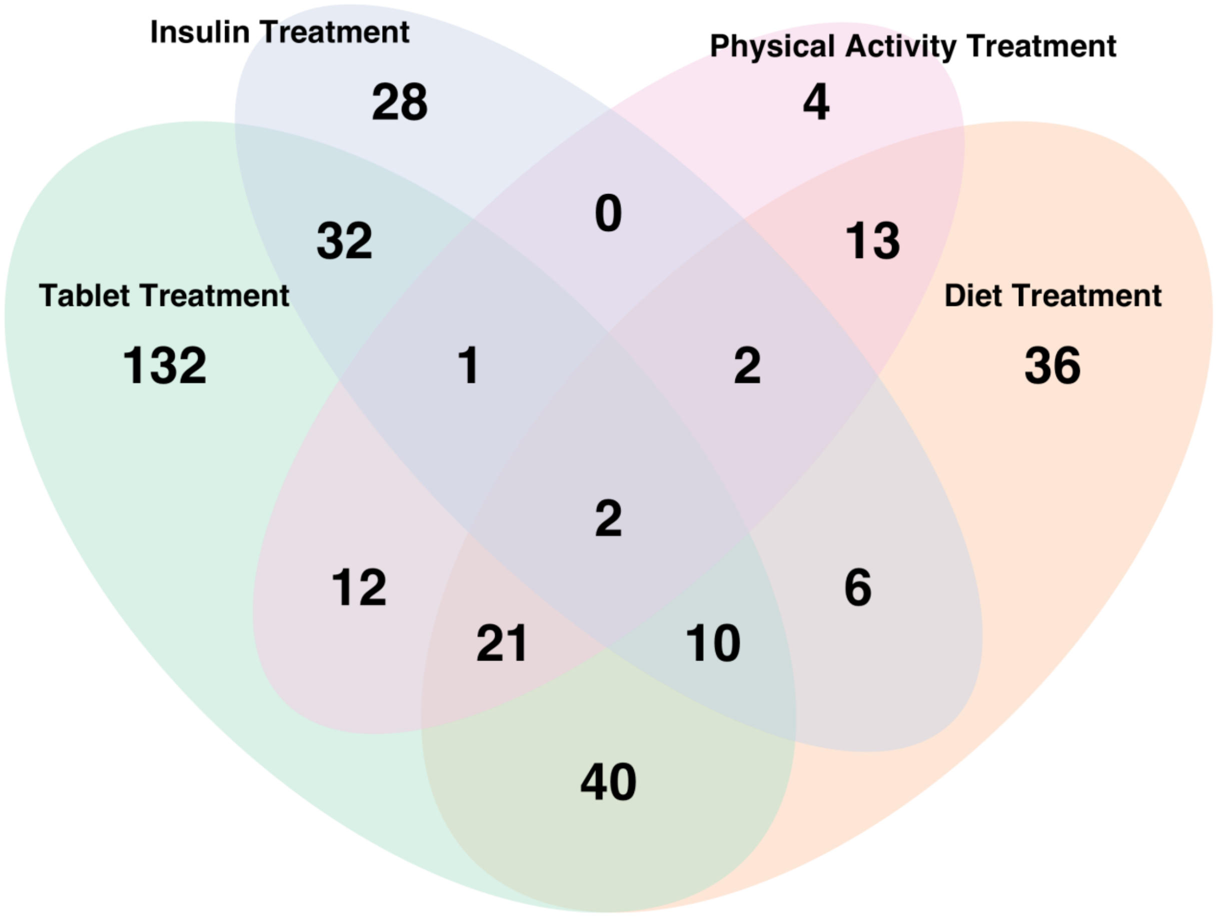

Supplementary Figure 10. Counts of treatment types for individuals with diabetes. Most individuals with a doctor diagnosis of diabetes were exclusively on tablet treatment, while others were on some combination of insulin, diet and/or physical activity.

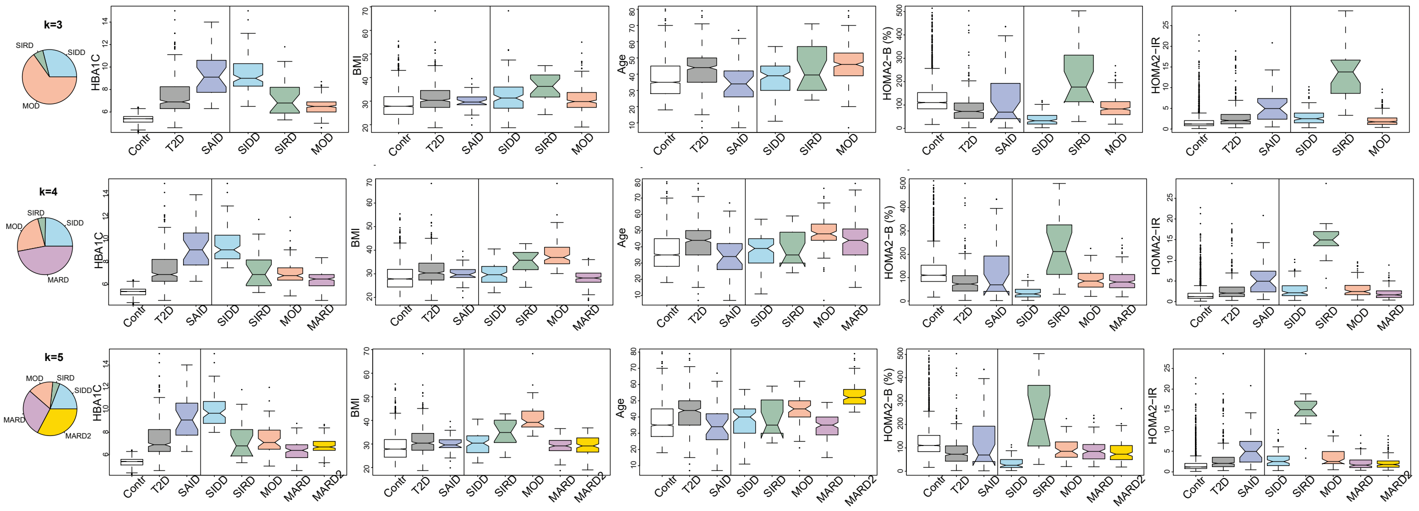

Supplementary Figure 11. Changes in cluster distribution for different k. Some changes can be observed in the cluster assignments when clustering QBB data using different k (3,4, and 5). The most prominent change is seen in the split of the MOD cluster to discriminate between older and younger individuals. SAID and control data were not included in the clustering but are visualized for comparison with T2D and the individual clusters. Contr: Controls (N=1,735 individuals), T2D: Type 2 Diabetes (N=420 individuals), SAID: severe autoimmune diabetes (N=29 individuals), SIDD: Severe Insulin Deficient Diabetes (N=93 individuals), SIRD: Severe Insulin Resistant Diabetes (N=17 individuals), MOD: Mild Obesity-related Diabetes (N=89 individuals), MARD: Mild Age-related Diabetes (N=177 individuals). Data in boxplots are presented as follows: lower and upper whiskers represent the minima and maxima respectively, box centers represent the median values, bounds of boxes represent the first and third quartiles, notches represent the 95% confidence interval of the median, and dots represent outliers.

Supplementary Figure 12. Data-driven clustering based on Gaussian mixture models. Models are estimated by the expectation-maximization (EM) algorithm initialized by hierarchical model-based agglomerative clustering. The optimal model and number of clusters was determined using the Bayesian Information Criterion (BIC) for EM. The top 3 models based on the BIC criterion, were k=3, k=4, and k=5. The spherical and diagonal models fitted in the EM phase of clustering are shown above. The three-character code represents the volume, shape, and orientation. The codes represent the following multivariate mixture models, "EII": spherical, equal volume, "VII": spherical, unequal volume, "EEI": diagonal, equal volume and shape, "VEI": diagonal, varying volume, equal shape, "EVI": diagonal, equal volume, varying shape, "VVI": diagonal, varying volume and shape, "EEE": ellipsoidal, equal volume, shape, and orientation, "VEE": ellipsoidal, equal shape and orientation, "EVE": ellipsoidal, equal volume and orientation, "VVE": ellipsoidal, equal orientation, "EEV": ellipsoidal, equal volume and equal shape, "VEV": ellipsoidal, equal shape, "EVV": ellipsoidal, equal volume, "VVV": ellipsoidal, varying volume, shape, and orientation.

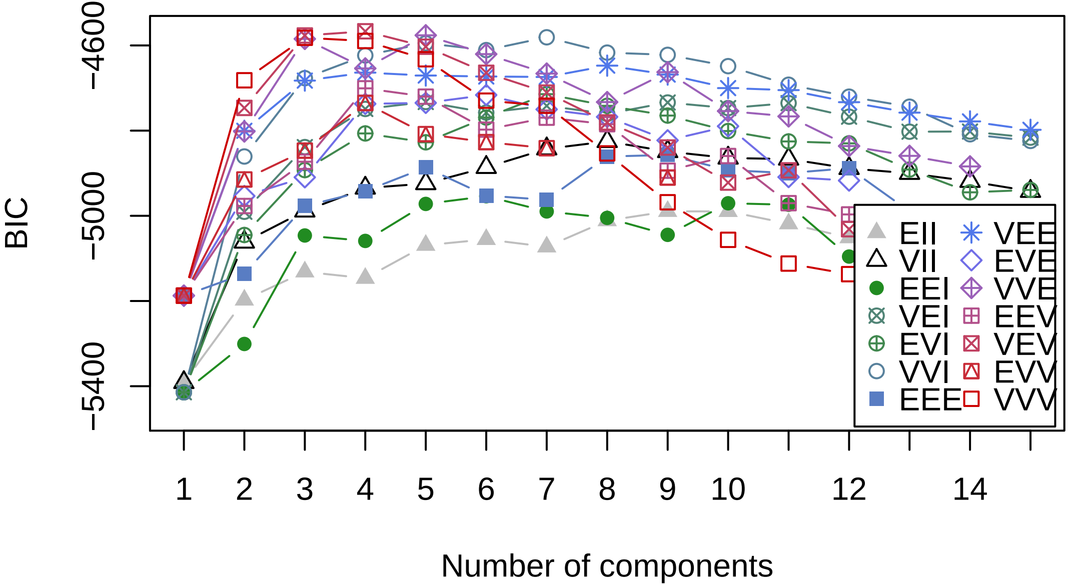

Supplement: Supplementary file 1 — Supplementary Information [file 41467_2022_34754_MOESM1_ESM.pdf]
