# Supplementary material for: Metabolic and proteomic signatures of type 2 diabetes subtypes in an Arab population
Source: Nat Commun. 2022 Nov 19;13:7121. doi: 10.1038/s41467-022-34754-z (PMC9675829; doi:10.1038/s41467-022-34754-z)
Supplement: Supplementary file 3 — Description of Additional Supplementary Files [file 41467_2022_34754_MOESM3_ESM.pdf]

Supplementary Data 1: Box plots for all protein levels associated with a T2D specific cluster. Data in boxplots are presented as follows: lower and upper whiskers represent the minima and maxima respectively, box centers represent the median values, bounds of boxes represent the first and third quartiles, notches represent the 95% confidence interval of the median, and circles represent outliers.

Supplementary Data 2: Box plots for all metabolite levels associated with a T2D specific cluster. Data in boxplots are presented as follows: lower and upper whiskers represent the minima and maxima respectively, box centers represent the median values, bounds of boxes represent the first and third quartiles, notches represent the 95% confidence interval of the median, and circles represent outliers.

Supplementary Data 3-21: Provided in one Excel file.
SD3: Cluster coordinates in QBB vs ANDIS for the four T2D subtypes.
SD4: T2D-protein associations.
SD5: Metabolite-T2D associations.
SD6: Replication of metabolite-T2D associations in QMDiab.
SD7: Cluster-specific significant proteins.
SD8: Cluster-specific significant metabolites.
SD9: Effect of confounding on cluster-specific proteins.
SD10: Effect of confounding on cluster-specific metabolites.
SD11: Cluster-specific metabolites correlation to diet and microbiome.
SD12: Replication of subgroup-specific proteins in AGES.
SD13: Replication of cluster-specific proteins from the IMI-RHAPSODY study.
SD14: Replication of subgroup-specific proteins in ANDIS.
SD15: Replication of cluster-specific metabolites from the IMI-RHAPSODY study.
SD16: Numbers and percentages for drugs taken by the T2D cases versus controls according to ATC anatomical main groups.
SD17: Number/percentage of drugs taken by ATC therapeutic subgroup for the 420 T2D QBB participants versus 1,735 controls.
SD18: Cluster-specific significant drugs.
SD19: Study demographics for training and testing sets.
SD20: Protein Information.
SD21: Metabolite Information.
